# Small Business Property Tax Reductions and Firm Productivity

**DOI:** 10.1007/s11187-023-00768-0

**Published:** 2023-05-09

**Authors:** Karolis Matikonis, Matthew Gobey

**Affiliations:** 1 Queen’s Management School, Queen’s University Belfast, Northern Ireland, UK; 2 Future Economies Research Unit and Department of Economics, Policy and International Business, Manchester Metropolitan University, Manchester, UK; 3 Present Address: Lochlann Quinn School of Business, University College Dublin, Dublin, Ireland

**Keywords:** Productivity, Business rates, Policy, Taxation, Small business, Applied machine learning, C55, D22, H23, H25, D24, O43

## Abstract

We investigate the productivity spillovers from the UK government’s decision to use extensive property tax reductions as a key instrument to stimulate innovation in smaller businesses and drive local growth. To capture the complex interaction and clustering of hierarchical effects, we apply non-parametric Random Effects Expectation Maximisation algorithm that complements more standard econometric estimators, namely matching to control for endogeneity and control functions to estimate total factor productivity. These approaches enabled us to incorporate various contextual configurations in comparing the recipients of these reductions to non-recipients with regard to productivity, in which the UK has experienced a considerably worse performance than its peers since the great recession. Contrary to policy assumptions and business community expectations, we show that generic tax reductions, when significant, are mostly associated with lower productivity and thus have been unsuitably chosen as a policy mechanism to stimulate productivity growth. We further show how instruments that are not built for causality could be beneficial for policy evaluation.

**Supplementary Information:**

The online version contains supplementary material available at 10.1007/s11187-023-00768-0.

## Abstract (plain language summary)

Reductions in small business property tax will not solve UK productivity problem. We investigate the productivity spillovers from the UK government’s decision to use extensive property tax reductions as a key instrument to stimulate innovation in smaller businesses and drive local growth. We incorporate various contextual interactions in comparing the recipients of these reductions to non-recipients with regard to productivity, in which the UK has experienced a considerably worse performance than its peers since the great recession. Contrary to policy assumptions and business community expectations, we show that generic tax reductions, when significant, are mostly associated with lower productivity and thus have been unsuitably chosen as a policy mechanism to stimulate productivity growth. Thus, the principal implication of this study is that instead of generic tax incentives based on building and land value, policy should be more nuanced and targeted, enabling the inclusion of finer-grained characteristics. We also contribute to methodology development by showing how configuration approaches, centring on non-linearity and equifinality, could be enabled through the application of machine learning to study organisations.

**Supplementary Information:**

The online version contains supplementary material available at 10.1007/s11187-023-00768-0.

## Introduction 

Advanced economies around the world are suffering from weaker levels of productivity growth post-2008 recession, but the UK’s poor performance is particularly pronounced (OECD, 2020). Productivity growth had averaged 0.3% from 2008, compared to 2.3% in the previous decade, and this gap is widening when compared with other advanced economies (HM Treasury, 2020). In this context, UK governments have enacted extensive commercial property tax reductions for smaller firms to incentivise investment, leading to productivity improvements and local growth (HM Treasury, 2016, 2017). Over the past decade, these tax reliefs have increased eightfold and have now removed approximately one-third of businesses from local tax bases, despite the concerns related to the lack of justification, mistargeting and the subsequent capitalisation of these reductions (Matikonis, 2020, 2022). This paper aims to empirically explore whether the effects of commercial property tax reductions on productivity vary and interrelate with firm, place, industry and policy characteristics. We use configurational approaches to delineate whether this expensive (more than £16bn to 2022, HM Treasury, 2017) policy works or whether we need more feasible delivery mechanisms.

The contested (e.g. Holtz-Eakin, 2000) policy narrative justifying these substantial tax subsidies rests on supply-side efficiency arguments. The subsidies are meant to overcome funding distortions for smaller firms, which face tighter financial constraints than larger ones. Freed from these constraints, it is assumed that firms invest and innovate (Shane, 2009). They in turn become more efficient and productive as they employ more qualified staff, better technology or improve their management, enabling catch-up with their larger competitors.

Nevertheless, this superficially plausible ‘one size fits all’ logic omits vital contextual location, market and industry factors. For instance, the delivery mechanism fails to account for the divergence between the statutory and economic incidence of taxation and the subsequent uneven appropriation of reliefs by property owners (Hilber, 2017; Matikonis, 2020, 2022). For those in receipt of funds, little consideration is given to encouraging them to invest in productivity improvements. Numerous factors, such as staff shortages (Bennett & McGuinness, 2009), investment uncertainties (Bloom et al., 2018) or demand-pull innovation (Piva & Vivarelli, 2007), could interact in limiting productivity gains.

Papers in SBEJ have been integral in contributing to our knowledge base of small business taxation, in particular, its effects on entrepreneurship (Baliamoune-Lutz & Garello, 2014; Bennett, 2021; Bruce & Mohsin, 2006; Ferede, 2021; Venâncio et al., 2022) with findings indicating the detrimental impact of taxation, including to productivity (Romero-Jordán et al., 2020). Although this is generally supported by literature on tax incentives (Liu et al., 2019; Sterlacchini & Venturini, 2019; Venâncio et al., 2022), Gobey and Matikonis (2021) show that this is not the case for the UK, where they find that small business property tax reductions do not produce additional employment.

We build on their study, but instead of employment, we focus on productivity when comparing the recipients of these reductions to non-recipients. Informed by the configurational approaches constructed from the complexity theory that rejects the ‘one size fits all’ approach to studying organisations (Meyer et al., 1993), we also account for vital contextual location, market and industry factors with the use of the novel Sela and Simonoff (2012) and Fu and Simonoff (2015) Random Effects Expectation Maximisation (RE-EM) decision tree algorithm.^1^ We show how these non-parametric techniques, known as regression trees, enable us to visualise and identify complex hierarchical relationships when analysing whether recipients of deep reductions in property taxes are associated with higher total productivity.

We visualise the significant levels of total factor productivity depending on the depth of the relief and other finer-grained characteristics, including location and industry-specific indexes. Our results suggest that when significant, the reductions in property taxation over sustained periods are associated with lower productivity levels, but this relationship interacts with numerous location, market and industry factors. Thus, we suggest that a nuanced and targeted policy incorporating an understanding of local drivers and constraints could be more fruitful than the current generic policy based on building and land value.

We structure the rest of the paper as follows. In Section 2, we summarise the UK property tax regime and its policy context. In Section 3, we briefly introduce configurational approaches. We then discuss the data, modelling and approach to estimation in Section 4. In Section 5, we present our findings, and we set out our conclusion in Section 6.

## Small Business Rate Reliefs

The Small Business Rate Reliefs (SBRR) are substantial reductions to the non-domestic property taxation receipts, more widely known as business rates (BRs), targeted at small businesses. These tax reliefs (or subsidies) were first introduced in Scotland in 2003, England in 2005 and Wales in 2007. Initially, they were temporary reliefs, but these and subsequent deeper reductions have become permanent over time, despite little evidence that they have supported local business or growth (Gobey & Matikonis, 2021).

BR is a substantive high-profile UK tax instrument which raises the equivalent of around half the revenue raised from corporation taxes and places a great cost on businesses.^2^ It was reformed as a centralised tax instrument, with powers removed from local councils in 1990 as part of the wider reform of local council funding, which included the infamous poll tax. Local councils have consequently had minimal ability to finesse the local application of the tax and associated reliefs to meet heterogeneous local needs. The SBRR are then centrally defined homogeneous (within each separate nation) and unconditional revenue reductions, which in England alone, up to 2022, have been forecast to reduce revenue by £16bn (HM Treasury, 2017). Moreover, the reductions introduced in 2017 reduced the liability for 900,000 firms, of which 600,000 saw their liability reduced to zero (HM Treasury, 2017). These are from an approximate total population of two million firms and as such revenues are increasingly dependent on a narrowing set of larger firms.

The calculation of a firm’s BR liability is based on an annually set multiplier linked to the rateable value of a property, which is estimated on a 5-year cycle by the Valuation Office Agency. Rateable values do not include such characteristics as sector, turnover or employment, leading to the mistargeting of SBRR (Matikonis, 2020). Instead, the estimation is solely based on land and buildings. The valuation includes certain categories of installed capital integral to a building (e.g. bicycle sheds to furnaces) which consistently leads to high numbers of appeals. The inclusion of capital in the tax base provides a further channel through which BR could affect productivity.

SBRR, depending on the nation and period, reduce the BR obligation in steps or sets a maximum tax reduction, which then tapers linearly.^3^ For example, in England, for 2010–2017, locations with a rateable value up to £6000 had a 100% reduction, which then tapered in a linear pattern to zero at £12,000 (this became the 100% threshold from 2017). We calculate the precise individual firm level of reliefs, and this process is explained in Appendix 1. Furthermore, the differential in rates faced by small and larger businesses is widened by the partial funding of the reliefs through a higher rate (multiplier) on premises with a rateable value above the qualifying SBRR threshold, but this contribution has not kept pace with the scale of reliefs (Gobey & Matikonis, 2021).

The issues with the mechanisms behind the overall BR system and SBRR have already been reviewed by Matikonis (2020, 2022), who voiced concerns about the lack of justification, mistargeting and subsequent capitalisation. The latter is because of the failure to account for the divergence between the statutory and economic incidence of taxation. This is fundamental in this context as the property occupier rather than the property owner faces the statutory responsibility to pay BR. The first to establish the incidence of UK property taxes was Fraser (1987). If we applied his reasoning to SBRR, we would expect SBRR to be received by occupiers because of the lag in rent review, but only in the short term. Property owners would later receive SBRR through increased rents and capital gains. Having said that, the pace and degree of this will not be uniform (Hilber, 2017) but rather dependent on the relative elasticities of property supply and demand conditions, amongst other factors.

Interacting with expectations on capitalisation is the assumption that firms would necessarily invest any funds released by the SBRR. Given the heterogeneous nature of the recipient firms,^4^ there are many factors which could interact with productivity gains. Some firms could potentially allocate extra resources towards improving the organisational environment, organisational capabilities, types of innovation, or external knowledgebase, which were found to be dominant in researching SME productivity, as summarised by Owalla et al. (2022).

Then again, some firms could also experience the absence of any demand-side stimulus to overcome firm investment (profit) uncertainties (Bloom et al., 2018) or generate demand-pull innovation (Piva & Vivarelli, 2007), which is more typical in small firms. These could be influenced by the degree of irreversibility in capital sunk costs (Carruth et al., 2000; Guceri & Albinowski, 2021), for which there has been no reduction in risk aversion (Appelbaum & Katz, 1986; Bianco et al., 2013). In this context, some firms will alternatively increase mark-ups or pass tax reductions to consumers to gain or stabilise market share amongst similar firms that have not received SBRR. These are only a few reasons amongst many why we could not expect uniform linear relationships when modelling the effects of SBRR on productivity.

## Configurational Approaches

Instead of uniform linear relationships, we more realistically anticipate complex interactions that influence changes in productivity. From the theoretical perspective, our reasoning thus directs us to the configurational approaches built from the complexity theory that rejects the ‘one size fits all’ approach to studying organisations (Meyer et al., 1993).

Configurational approaches have been defined as an analysis of a ‘multidimensional constellation of conceptually distinct characteristics that commonly occur together’ (Meyer et al., 1993). The theory emphasises heterogeneity in organisations and views them as a combination of factors that particular organisations share. The relationships are non-linear, and two or more configurations may be equifinal in affecting the outcome variable (Meyer et al., 1993). The conceptualisation based on configurational approaches thus enables us to depart from strictly linear relationships. It supports the notion that ‘variables found to be positively related in one configuration may be unrelated or even inversely related in another’ (Meyer et al., 1993: 1178). In this way, we can deviate from the not-so-useful concept of ‘average entrepreneur’, as discussed in Newbert et al., (2022:4) and enable modelling realities using complex configurations that are necessary to understand the phenomena (Woodside, 2014).

The concerns of existing equifinality, multi-finality and non-linearity in relationships triggered many papers in SBEJ to adopt the reasoning of configurational approaches, with several publications embracing this theory to explain entrepreneurship related phenomena, including gender (Sperber & Linder, 2019), crowdfunding (Huang et al., 2022), attention deficit and hyperactivity disorder (Hatak et al., 2021), ecosystems (Wang et al., 2023) and firm performance (Su et al., 2011).

## Empirical Methodology

### Data

We base our analysis on the UK ONS Annual Respondents Database X, first released in July 2016 combined with Business Structure Database and Prices Survey Microdata. Descriptive statistics of raw data are available in Appendix 2.

The Annual Respondents Database X combines two existing surveys, the Annual Business Inquiry (1998–2008) and the subsequent Annual Business Survey (2009–2014), which firms’ representatives are legally required to complete, producing high response rates. It is a complex stratified sample across size, sector and region. The sample framework is constructed using administrative data on employment and turnover from PAYE^5^ and VAT-registered firms. Importantly for our purposes, it captures information at both the enterprise and local unit levels. We limit the sample to firms that have only one local unit because businesses, with some exceptions, have to use only one property to receive SBRR. We also need to calculate firm’s rateable value and SBRR from the survey reported BR (see Appendix 1).

We combine this data source with the Business Structure Database to acquire the observations from smaller firms that were not included in the Annual Respondents Database X.^6^ The Business Structure Database contains an annual release of a small number of critical variables on all UK registered firms and is complementary to the above business surveys.

The Annual Respondents Database X and Business Structure Database do not directly provide controls for the input price changes that we require for the estimation of productivity. To control for omitted price bias (as defined by Van Beveren, 2012), we do not use the typical approach of employing the inherently biased general gross domestic product, but use the Prices Survey Microdata data, which contains more accurate regional and sector level prices. We devalue to 2016 prices.

### Estimation Strategy

To illustrate whether the non-domestic property tax reliefs have any impact on productivity, we could simply estimate:

1 $${\omega }_{it}=\beta {\alpha }_{it}+{e}_{it}$$

where ${\omega }_{it}$ is the productivity of establishment $i$ at time $t$ and error term, ${e}_{it}$, that captures the demand shock ${\rho }_{ij}$ in reduced form. The main parameter of interest is $\beta$ capturing the effect of any relief, ${\alpha }_{it}$.

Standard estimators such as Ordinary Least Squares would not yield a consistent estimate of $\beta$ because establishment-level characteristics are unlikely to be independent of each other or local characteristics. Following Gemmell et al. (2019), the first step in addressing the feasible complex relationships is to exploit our large representative dataset to recreate the conditions of a quasi-natural experiment^7^ in which firms that receive SBRR are matched to similar single-unit firms which do not. Our large dataset enables us to use a wide range of observable establishment level characteristics, namely materials, age, investment, rent, output per employee, employment, sector, legal status, turnover and gross value added, to produce matches. The dependent variable is a dummy taking a value if one for those firms that received the relief at least twice between 2003 and 2015 and zero otherwise.

However, instead of the more popular Propensity Score Matching, we match using Coarsened Exact Matching. It uses a more efficient fully blocked randomised experiment rather than attempting to approximate a completely randomised experiment as applied in the Propensity Score Matching, which was found to increase imbalance, model dependence and bias (King & Nielsen, 2019). The SBRR recipient firms are matched 1 year prior to the introduction of SBRR or, in the case of young firms, on their first observable year to corresponding non-recipient firms.

Separate matching is performed for each year starting with 2002, with the non-recipients being excluded from further matching if they matched previously. Thus, most of the matching was performed on 2004 data, 1 year prior to the SBRR introduction in England. This produced a final dataset for the years 2000 to 2015 of 15,047 observations for 1092 firms, 546 SBRR recipients matched to 546 firms which had never received the relief, yet had similar characteristics. To describe the reduction in imbalance after matching, as per Iacus et al. (2009) recommendations, we estimate the ${\mathcal{L}}_{1}$ statistic that includes imbalance with respect to joint distribution and all interactions between recipients and non-recipients. The matching produced a substantial reduction in imbalance with ${\mathcal{L}}_{1}$ decreasing from 0.776 to 0.592 for 2004.^8^

We then expand our specification with variables that provide a more realistic setting for the analysis of heterogeneous establishments. For simplicity, we classify our independent variables as time-varying establishment-specific variables, ${Z}_{it}$, whilst establishment-fixed effects are captured by the intercepts, ${f}_{i}$. That is^9^:

2 $${\omega }_{it}=h\left({\alpha }_{it},{Z}_{it},{f}_{i}\right)={\beta }^{a}{\alpha }_{it}+{\beta }^{b}{Z}_{it}+{f}_{i}+{e}_{it}$$

This specification still does not fully identify the complex groupings of firms. Building on the configurational approaches literature (Meyer et al., 1993), we imply that a number of variables are unlikely to be additively separable and permit us a priori to establish a clear mechanism through which the policy affects productivity. Controlling for all feasible interactions would produce a complex number of coefficients, possibly even a unique set of coefficients for each firm, ${\beta }_{it}^{a}$ and ${\beta }_{it}^{b}$. Such estimates would be difficult to interpret given that we cannot establish a defendable identification strategy. The estimates are also likely to be biased, especially for large datasets (Gandomi & Haider, 2015).

More standard estimators, such as mixed-effects or difference-in-difference estimators, could provide some insight into uniform relationships. However, these relationships are unlikely to be uniform but interaction-dependent because of inherent mistargeting and the subsequent uneven capitalisation amplified by different decision choices. Our reasoning is supported by the results from the difference-in-difference estimator in Table 1 that finds consistently negative but only somewhat significant results, which we further discuss in Section 4.5.

**Table 1:** Estimates of treatment effect with the difference in difference regressions

Variable	(1)	(2)	(3)	(4)	(5)	(6)	(7)	(8)
Treatment and time	− 0.001 (0.002)	− 0.001 (0.002)	− 0.001 (0.002)	− 0.001 (0.002)				
Extent, treatment and time					− 0.006 (0.003)*	− 0.006 (0.004)	− 0.006 (0.003)*	− 0.006 (0.004)*
Controls	No	No	Yes	Yes	No	No	Yes	Yes
Firm-fixed effects	Yes	Yes	Yes	Yes	Yes	Yes	Yes	Yes
Year dummies	Yes	Yes	Yes	Yes	Yes	Yes	Yes	Yes
Sector cluster	Yes	Yes	Yes	Yes	Yes	Yes	Yes	Yes
SE correction	No	Yes	No	Yes	No	Yes	No	Yes

*refers to a 90% significance level

We, thus, require an estimation strategy capable of identifying complex groupings of firms. Empirical studies based on configurational approaches in SBEJ primarily used either standard statistics (Su et al., 2011), limited by assumptions of linearity, or more advanced fuzzy-set Qualitative Comparative Analysis (Hatak et al. 2021, Sperber & Linder, 2019, Wang et al., 2023). This method was found to be prone to subjective bias, require extensive data calibration and heavily rely on prior knowledge (Liu et al., 2017).

Instead, we draw on decision trees that have already been successfully applied in the real estate context with Feldman and Gross (2005) as well as in growth determinants with Tan (2010) and more recently in conjunction with configurational approaches (Graham & Bonner, 2022), who also discussed the advantages of these approaches. They stressed their ability to handle large datasets with various data types, missing values and outliers as well as their ability to capture interrelationships between variables in different parts of the measurement space, which is essential given the varying capitalisation and investment decisions. These approaches are, however, susceptible to overfitting (Cook & Goldman, 1984) and instability (Briand et al., 2009). These issues are addressed in Section 4.6.

### Model

We adopt a more recent extension than in Tan’s (2010) and exploit the RE-EM decision tree approach of Sela and Simonoff (2012) and subsequently, Fu and Simonoff (2015). This technique combines estimates from fixed and random effect trees to discover the complex groupings of firms. The random-effects element accounts for the constant differential firm-level factors, whilst the decision tree allows the data to discover the complex groupings of firms and their different levels of productivity without imposing a complex parametric structure.

The RE-EM approach assumes that neither the random effects nor the fixed effects are known and alternates between estimating the regression tree, assuming that the estimates of the random effects are correct, and estimating the random effects, assuming that the estimates from the regression tree are correct. A brief introduction to decision trees is offered in Appendix 4, and a more detailed explanation of the mechanisms of RE-EM is available in Sela and Simonoff (2012).

The RE-EM model is:

3 $$\begin{array}{c}{\omega }_{it}={Z}_{it}{b}_{i}+f\left(.\right)+{\varepsilon }_{it}, i=1,\dots , I t=1,\dots , n \\ \left(\begin{array}{c}\begin{array}{c}{\varepsilon }_{i1}\\ :\end{array}\\ :\\ {\varepsilon }_{in}\end{array}\right) \sim N\left(0,{R}_{i}\right),\\ \begin{array}{c}{b}_{i}\sim Normal(0,D)\\ f\left(.\right)=f{\left(\sum_{j=0}^{4}\left({SBRR}_{t-j}\right), {\rho }_{it},{a}_{it},{r}_{it},{s}_{it}, P{S}_{it}, P{D}_{it}, HH{I}_{it},R\&{D}_{it},HG{F}_{it}, {FO}_{it},{IO}_{it}\right)}\end{array}\end{array}$$

The dependent variable, ${\omega }_{it}$, is our bootstrapped estimate of productivity, as discussed below for each firm *i* in period *t*. $Z$ is a matrix of independent variables which may vary over time and firms and ${b}_{i}$ is the vector of random effects. *f(.)* contains the same variables as *Z*, although they can differ, which we use to estimate the fixed effects via the decision tree.

Within *f(.),* we define ${\alpha }_{it}$ as $g\left(\sum_{j=0}^{4}\left({SBRR}_{t-j}\right), {\rho }_{it}\right)$, i.e. *SBRR* and four lags to capture medium-term effects and account for the periodicity of the reliefs. We complement these variables with the dummy variable $\rho$ to capture the initial^10^ effects of receiving any relief or uplift in relief, irrespective of level.

We include the broad sectors (*s*) of wholesale, catering, construction, production, property, retail and other services and foreign ownership. The Office of National Statistics (2017) calculates that UK firms receiving foreign investment have 74% higher productivity than those which do not. As such, we include the dummy variable $FO$ that takes a value of 1 for firms with a foreign majority owner to account for any systematic effects. In this era of concerns about complex ownership structures and use of complex taxation schemes, we also use the variable $IO$ to denote a foreign country registration of the firm’s immediate parent firm, as also employed by the ONS. This can be different from $FO,$ which denotes the ultimate country of the owner.

We also control for firm age (*a*), whether it is high growth (*HGF*) and Research and Development (*R&D*) active. Since the pioneering work of Griliches (1979), productivity models^11^ have considered technological spillovers to be a side product of R&D activities, as such we control for whether a firm intends to undertake R&D within the next 2 years.

*HGF* is a dummy taking the value of 1 in the years in which a firm meets the Eurostat-OECD (2007) definition, namely average annualised growth in employment greater than 20% per annum, over a 3-year period with initial employment not lower than 10.

Beyond firm-level effects, we explore important national, regional and small (two-digit postcode) location and industry specific effects. We include the regions and nations (*r*) of Wales, Scotland, North East, North West, Yorkshire and Humberside, East of England, East Midlands, West Midlands, London, South East and South to control for fixed location effects. Given our access to detailed firm-level microdata, we include finer spatial and time-varying indices for Jacob production diversity (*PD*) and Marshall production specialisation (*PS*), within small two-digit postcode areas, relative to national SIC (2003) two-digit industry output. Finally, we control for industry concentration at the national level via a Herfindahl–Hirschman Index (HHI).

Various specifications of these indexes may influence the results and their interpretation. In terms of PS and PD, we wanted to ensure that the measures complement each other and can coexist in one equation. Thus, we follow the specification of Modrego et al. (2015), who derive PS as region and sector-specific, whilst PD as region-specific, enabling them to coexist (Van der Panne, 2004). In terms of HHI, we followed the commonly applied design (e.g. in Fairlie et al., 2023), which estimates HHI by squaring each firm’s market share and then summing the resulting numbers.

The PS index captures relative industrial clustering effects. For example, the agglomeration may enable the creation of better labour pools, supplier services or the spillover of incremental process and product innovations. SBRR may interact with these local factors by reducing the assumed financial barriers to adoption or creation of incremental changes. That said, we may also observe increased competition for specific types of premises and a more rapid capitalisation of any tax reliefs.

We calculate the PS index in line with Modrego et al. (2015), Feldman and Audretsch (1999) and Paci and Usai (1999).^12^ However, our detailed data enables us to enhance the accuracy of the index by using firm turnover rather than employment to create the index. This produces a less noisy control for productivity than employment and a far more accurate perspective on the concentration and value of activity. The index is:

4 $${PS}_{i,j}=\frac{{~}^{{T}_{ij}}\!\left/ \!{~}_{\sum_{i}{T}_{ij}}\right.}{{~}^{{\sum }_{j}{T}_{ij}}\!\left/ \!{~}_{\sum_{i}\sum_{j}{T}_{ij}}\right.}$$

where* T* is industry *i* turnover in area *j*. We calculate the turnover of a given industry (*i*) in an area (*j*) as a proportion of all turnover in that area and then place it in relation to national turnover from the same industry as a proportion of national turnover.

We capture any local Jacob (Production) Diversity effects via an index based on the reciprocal of the Gini Coefficient as proposed by Paci and Usai (1999):

5 $${PD}_j=\frac2{(n-1)Q_n}\sum_{i=1}^{n-1}\;Q_i$$

where $n$ is the number of industries in region *j*, ${Q}_{i}$ is the cumulative turnover up to industry $i,$ then ordered by ascending size. The index, bounded by 0 and 1, increases with variety. Differently to HHI or PS, PD captures whether location, rather than the firm or industry, is at the centre of analysis and drives changes (Florida et al*.*, 2017). Innovation is aided by access to ideas and procedures that firms can copy or modify from a diverse set of industries or knowledge generating institutions within small areas or, given the positive correlation with urban areas, more diverse and stable demand. That said, at our two-digit postcode level, we will observe a substantial degree of variation even within urban areas.

The error term, ${\varepsilon }_{it}$, is assumed to be uncorrelated with the random effects and independent across observations. ${R}_{i}$ is a non-diagonal matrix to account for autocorrelation within firms.

### Dependent Variable: Total Factor Productivity

The most apparent first-order effect of SBRR is a reduction in investment in capital. Having said that, for some organisations, this could trigger expenditure in other areas that could be equally effective, including organisational environment, organisational capabilities, types of innovation, external knowledgebase, or even commercialisation that were found to be dominant in researching SME productivity, as recently summarised by Owalla et al. (2022). Considering this in connection with the ideas of heterogeneity in organisations from the configuration approaches literature, discussed in Section 3, we conclude that the concept of comparing total outputs relative to the total inputs used in the production of the output in the SBRR context could provide a fuller picture for our exploratory analysis than focusing on one specific outcome. We also depart from the commonly applied single-factor productivity measures, such as labour productivity, because multi-factor productivity measures better capture the changing trends in the working environment (Owalla et al., 2022) and avoid such limitations as the attribution of all increases of efficiency to one factor (Linna et al., 2010).

Total factor productivity is not directly observed from production functions and consequently needs to be extracted once the weighted sum of inputs has been estimated with controls for simultaneity and selection biases. We resort to control function approaches that are built to overcome these biases (Van Beveren, 2012). We employ Wooldridge’s (2009) approach, which builds on the work of Olley and Pakes (1996) and Levinsohn and Petrin (2003). His single-step GMM framework also overcomes more recent criticism directed towards the control function estimators failing to consistently estimate the labour coefficient in the first stage (Ackerberg et al., 2015). We thus estimate productivity assuming a Cobb Douglas functional form:

6 $${\omega }_{it}={e}^{{lnGVA}_{it}-{\beta }_{k}ln{K}_{it}-{\beta }_{l}ln{L}_{it}}$$

where ${\omega }_{it}$ is productivity of the *i*th firm in period *t*, ${lnGVA}_{it}$ is the firm’s logarithmic gross value added in order to simplify the model and eliminate intermediate inputs, $K$ is logarithmic capital and $L$ is logarithmic labour. To reduce selection bias, we averaged estimates from 1000 estimations with missing values replaced by the predictive mean matching with the key variables of unique firm identifier, year, turnover, employment, region, sector and legal status that have almost no missing data from the Annual Respondents Database X or Business Structure Database. For instance, if a firm had all observations but no data for 2005, we would impute the 2005 data 1000 times and produce 1000 datasets, which then were used to estimate 1000 separate models defined in Eq. (6).

### Difference in Difference Estimator

To compare the findings to more traditional approaches, we also conduct analysis with a more standard difference in difference estimator that has been widely employed in numerous recent publications in SBEJ (Amamou et al., 2022; Bailey, 2017; Biancalani et al., 2022; Liu et al., 2019; Dosi et al., 2012; Lewis, 2017) that show various specifications and empirical strategies of this approach. There seems to be no consensus on which strategies are preferred, with scholars trading increasingly restrictive assumptions with solutions to various issues, such as heteroscedasticity and autocorrelation.

We primarily follow the empirical strategy adopted in the recent study by Biancalani et al. (2022), who also offer a more detailed explanation of the methodology behind the estimator. Our difference in difference estimator thus departs from the standard specification in that the treatment variable is equal to 1 for firms receiving SBRR and only during years when they actually receive SBRR and 0 otherwise in models (1) to (4). In addition, we incorporate the extent of SBRR in models (5) to (8). We control for firm and time fixed effects in all specifications and cluster standard errors on sectors. We also include similar controls to those in *f(.)* in Eq. (3) in models (3), (4), (7) and (8). We estimate the treatment effect with the simple fixed effects panel regression in models (1), (3), (5) and (7), which we compare to estimates in models (2), (4), (6) and (8) that use Newey and West’s (1994) automatic bandwidth selection procedure to produce heteroscedastic and autocorrelation consistent estimation of the covariance matrix of the coefficient.

The results suggest that SBRR seems to have an adverse effect on some firms in terms of their productivity, but it does not alleviate a binding constraint for the average company. As reported in Table 1, we find consistently adverse treatment effects but these effects amongst firms seem to vary, as indicated by the relatively high error estimates in models (1) to (4) that resulted in insignificant coefficients. Once we include the extent of SBRR, the negative coefficients are greater, and the error terms are relatively smaller, resulting in a significant relationship but only at a 90% significance level in models (5), (7) and (8), indicating that the likelihood of companies receiving more relief to have lower productivity is greater. For instance, those with 100% SBRR have 0.6% lower productivity, with other variables keeping constant. This, thus, supports the need to explore these nuanced relationships further with such tools as RE-EM trees.

### Model Diagnostics

We also perform further model diagnostics to ensure that our model does not suffer from widely known issues. To overcome the issue of overfitting (Cook & Goldman, 1984), we impose a minimum number of observations in each final node. To test stability, we follow the Philipp et al. (2018) framework and implementation. The RE-EM trees achieved the highest stability score for this dataset, a median of 0.82. To assess the accuracy, we use tenfold validation, in which we compare the predictive capacity of our trees to other candidate algorithms. The RE-EM tree achieved a relatively high accuracy when compared to alternative methods. Root mean square error estimates indicate that the RE-EM tree achieved the highest accuracy, but that was not the case with mean absolute error, which accounts for extreme outliers. Based on this estimate, random forests slightly outperformed RE-EM tree. More detailed procedures and results are reported in Appendix 3.

## Findings

**Fig. 1:**
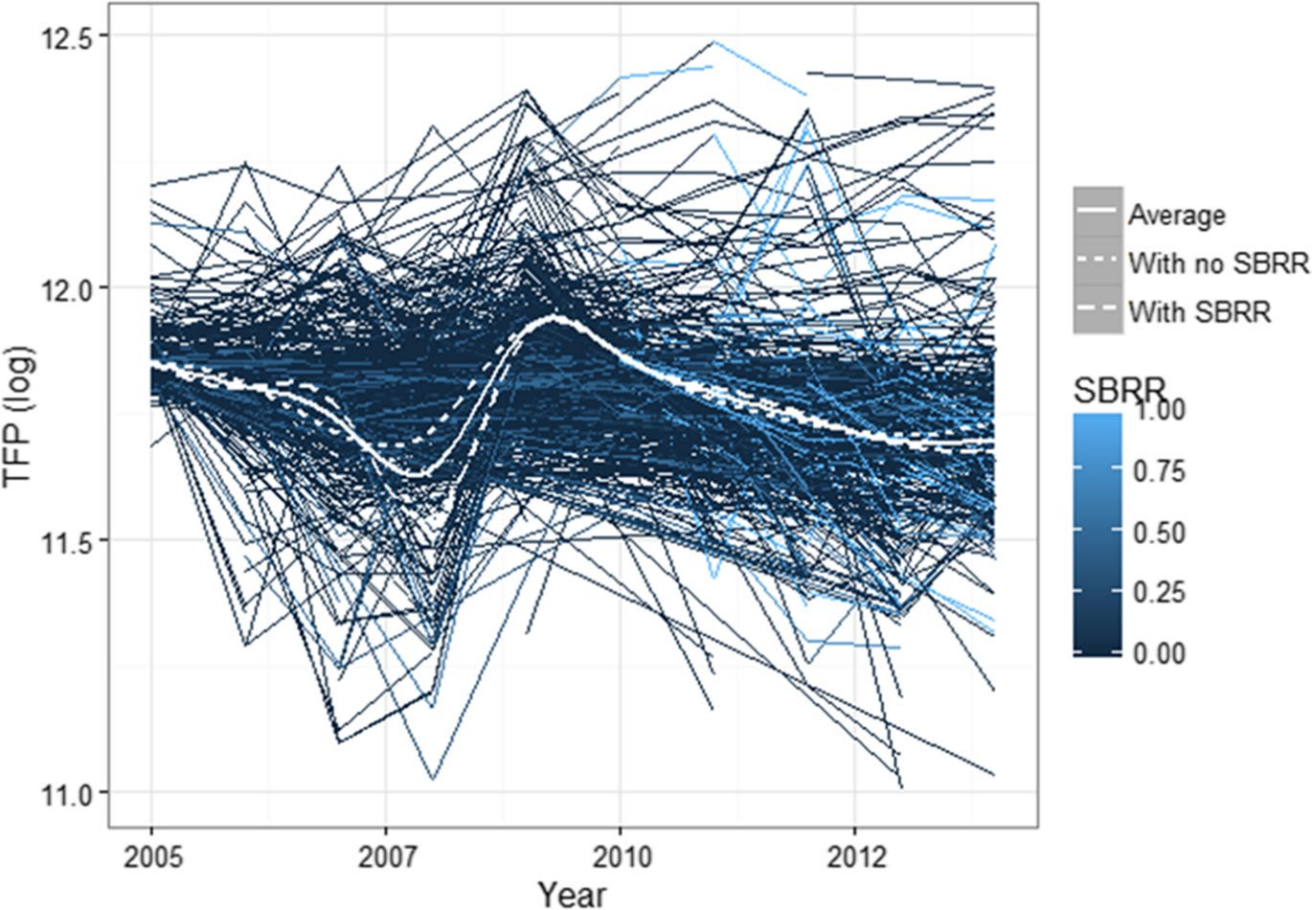
Estimated productivity 2005–2015 by SBRR

Without any controls, recipients of SBRR had on average 2.6% lower productivity in the matched sample and 2.7% lower productivity in the unmatched sample. The matching also reduced average productivity by 14.5%, indicating that possibly more productive larger firms were excluded from the analysis after matching. In Fig. 1, we further provide the firm-level evolution of estimated productivity during 2005–2015 and link this to receipt and degree of SBRR. We see a dramatic drop and rebound just after the start of the economic crisis in 2008. The average level of productivity was somewhat similar whether firms did or did not receive SBRR, although those in receipt of SBRR saw a steeper drop in 2008. We also see a weak identification of a negative trend, where the increases in reliefs are followed by the reduction in productivity. This is, however, to a lesser extent post 2008 recession, when reliefs became more generous post 2010. Overall, there is diverse and complex variation around the average levels of productivity amongst these carefully matched firms.

**Fig. 2:**
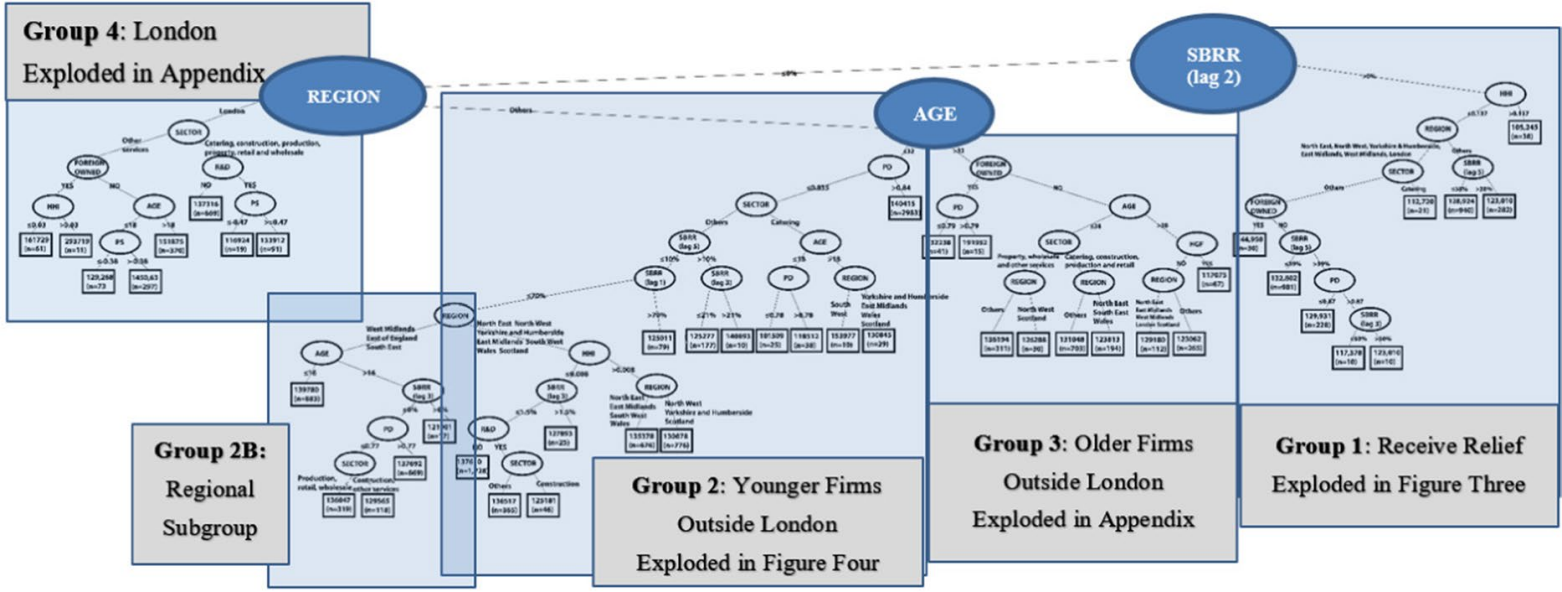
Productivity tree groupings

We present the full productivity decision tree estimates in Fig. 2, but given the number of significant groupings, we explode sections for more detailed analysis. The decision tree algorithm works by allowing the data to define ever more ‘pure’ groups of firms which explain variation in the dependent variable, in that the firms in the groups (nodes) are increasingly homogeneous. The final nodes give the number of firms in the group and the average level of productivity in terms of gross value added. The splits are not strictly hierarchical as variables can enter a branch more than once, but with different sub-values for points at which the data split.

**Fig. 3:**
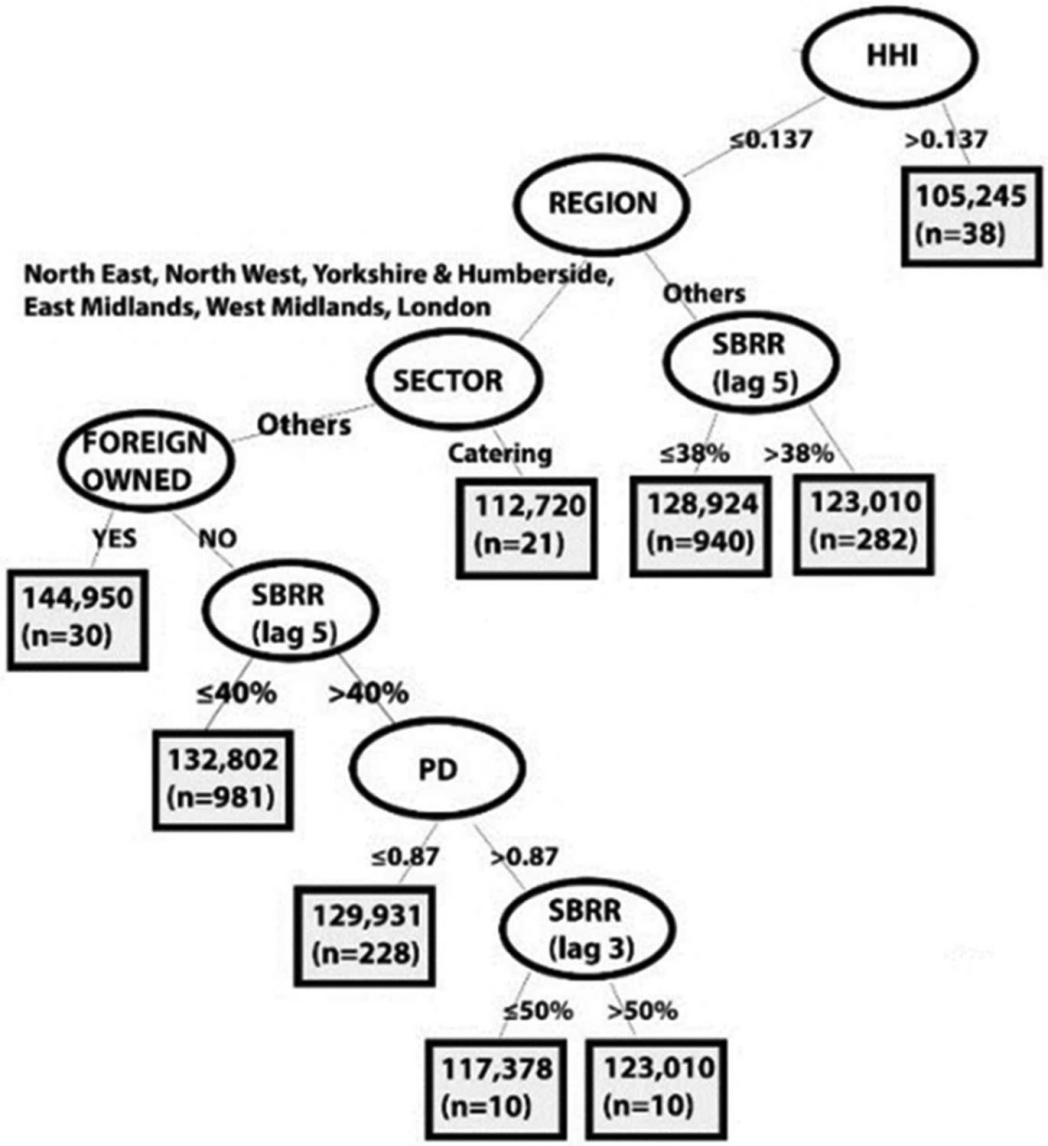
Productivity tree extract following firms receiving 2-year lagged SBBR

The principal split is on a 2-year lag of SBRR showing 12,502 observations with zero relief (left of the node in Fig. 2) and 2540 with some relief (right of the node). In Fig. 3, we follow the 2540 observations for firms receiving SBRR. The weighted average productivity of these observations is 6% lower than the other observations of firms not in receipt of SBRR. The first significant grouping along this branch is by region. In the Southern and Eastern regions of England, Wales and Scotland (others in Fig. 3), we identify a group of 1222 observations with 5-year lagged SBRR which divide on relief of 38%. Observations for the 940 firms in these regions receiving lower relief have higher productivity. The weighted productivity for these 1222 observations is lower than for observations in the Northern, Midlands and London regions of England, by more than 3%. In fact, for these English regions, the next significant node is also on 5-year lagged SBRR, which divides at a similar value of 40%. The pattern of productivity is the same, with lower productivity for the 248 observations from firms receiving higher levels of relief than the 981 observations with lower or no relief. We conclude from this branch that irrespective of region and sector (excluding catering in some regions), when significant, the greater extent of SBRR is broadly associated with lower productivity.

**Fig. 4:**
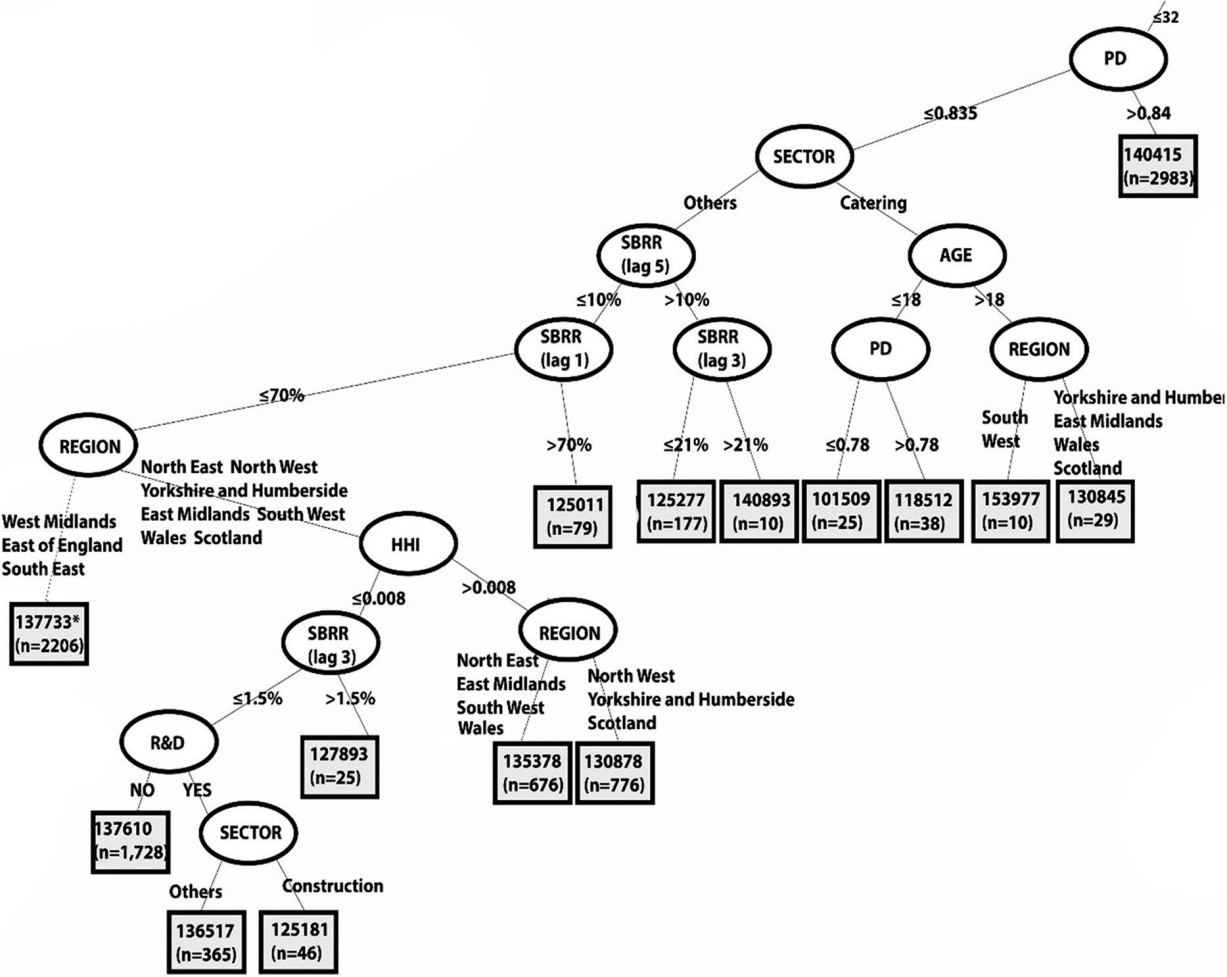
Productivity tree extract of younger firms not receiving 2-year lagged SBBR, outside of London (group 2)

Following the observations for firms which did not receive 2-year lagged SBRR, we find the specific characteristics of firms based in London differentiate them from observations in other regions. We firstly consider the 10,911 observations outside of London which next group on firm age, above and below 32 years of operation. In Fig. 4 (group 2 in Fig. 2), we follow the 9173 younger observations as these are influenced by SBRR. The 1738 observations from firms at least 32 years old (group 3 in Fig. 2) have no grouping on SBRR and ultimately the observations group by sector and then by region. The weighted productivity of these older observations is approximately 5% lower than that for the younger observations, but with significant variation. For 2,983 observations from younger than 32 years old SMEs in Fig. 4, we observe the split on high levels of local diversity, above 0.84 on the Jacobian production diversity (PD) index.^13^ The Jacobian PD hypothesis is that greater local diversity enables innovation. Here we find some support for this, as the weighted average productivity of these observations is approximately 3.6% higher than the productivity of others in two-digit postcode areas with a lower level of diversity. In the entire tree, we only find splits on Marshall Specialisation (*PS*) for areas of London.

Moving down the branch for the majority of firms, those with lower local output diversity (PD), we see, for all sectors except catering, that there is a complex division between long-term and short-term reliefs. In particular, observations with 5-year lagged SBBR below 10% and 1-year lagged SBBR below 70% are associated with productivity generally higher than those which have 5-year lagged relief above 10%. We do see some subsequent small divisions with similar productivity. Overall, we again find the recipients of greater SBRR are those with lower productivity.

Returning to the 1591 observations in London (group 4 in Fig. 2), we calculate a weighted average productivity five per cent higher than in the rest of the UK. This seems to be driven by a relatively small number of observations in sectors other than catering, construction, production, property, retail or wholesale. However, we find no association with SBRR.

Overall, our findings show that continued receipt of higher reliefs is associated with lower productivity. Other than SBRR, regional effects consistently explain variations in productivity, but we cannot say, except for London, that there is a simple dominant geographical pattern. Interestingly, across the tree, we find nearly 4000 observations in small areas with higher sectoral diversity (at least 0.78) have higher productivity than those with lower diversity. This suggests that the underlying institutional factors stimulating diversity could be a more logical policy target than generic tax reductions.

## Conclusions

This paper captured and visualised the significant effects of SBRR on productivity and showed how these interrelate with firm characteristics and location, market and industry dynamics. By doing that, it provided empirical evidence on the effects of mistargeting and capitalisation that were previously only theorised in Matikonis (2020, 2022). We discuss the main implications of the results from theoretical, practical and methodological perspectives.

### Contribution to Knowledge

Our paper extends the existing knowledge in SBEJ on how taxation affects small businesses. The consensus is that taxes are detrimental to entrepreneurship (Baliamoune-Lutz & Garello, 2014; Bennett, 2021; Bruce & Mohsin, 2006; Ferede, 2021; Venâncio et al., 2022), including productivity (Romero-Jordán et al., 2020) and tax incentives have positive effects (Liu et al., 2019; Sterlacchini & Venturini, 2019; Venâncio et al., 2022). Our results are contrary to these findings. This difference is likely to result from mistargeting and uneven capitalisation (as discussed in Matikonis, 2020, 2022), from which other than property tax instruments are less likely to suffer.

We find that these relationships are not uniform but highly dependent on the context, extending Hilber’s (2017) synthesis to non-domestic property. For instance, with regard to regional patterns, London has a higher average weighted productivity, but this is related to only a small number of extreme firms, not SBRR. In the rest of the UK, we find for approximately one quarter of observations that higher productivity is associated with greater small area diversity,^14^ particularly for younger firms. This finding is counter to Harris and Moffat (2017), who find agglomeration rather than diversity externalities is associated with higher productivity. This is likely to be because our approach accounts for the complex interaction effects of firm, place, industry and policy.^15^ Having said that, these findings on small area production diversity do not suggest the central government should focus on building local diversity across the UK, but rather focus should stay on further devolution, enabling the inclusion of finer-grained local dynamics to produce more successful local growth policies.

Our findings are also supplementary to the existing knowledge in SBEJ, which could add to the variety of the previous findings. For instance, previous studies found that taxation affects firm exits positively and entries negatively that was shown to be the case also for property taxes (Bennett, 2021). Similar findings were found by those that analysed other tax instruments, with results pointing to tax incentives increasing the number of entrants (Liu et al., 2019 and Venâncio et al., 2022). We, thus, suggest that tax incentives are likely to positively contribute to firm entries and reduce exits, but some of those new entrants or sustained firms will potentially become less productive, but this will depend on firm characteristics and location, market and industry dynamics.

### Methodological Contributions

We show how configuration approaches, centring on non-linearity and equifinality, could be modelled with decision trees. In this way, we deviate from the not-so-useful concept of ‘average entrepreneur’, as argued by Newbert et al., (2022:4), and model more realistic realities using complex configurations necessary to understand the phenomena (Woodside, 2014). The RE-EM trees, an extension of the decision trees, consider the relationship between independent and dependent variables to be not necessarily linear, enabling the capture of more complex relationships, whilst regression-based approaches fail to do that by centring on net effects instead of differences between groups (Douglas et al., 2020). Decision trees also do not suffer from other common regression-based assumptions, such as lack of multicollinearity, the distribution of errors and independence of observations (Douglas et al., 2020).

Furthermore, the paper showed how machine learning could be jointly applied with more standard econometric techniques to produce a finer-grained analysis when more standard estimators do not offer valuable insight. The more standard difference in difference estimator, reported in Section 4.5, suggested that SBRR has an adverse effect on some firms in terms of their productivity, but it did not alleviate a binding constraint for the average company. The RE-EM trees enabled us to extend these conclusions by departing from the average effect to uncovering the exact groupings of firms whose productivity was impacted by SBRR and showing how these interrelate with firm characteristics and location, market and industry dynamics.

### Implications for Policy and Practice

The study provided evidence that the current centralised policy of commercial property tax reductions does not work as an enable of growth. We need more feasible delivery mechanisms that support small businesses. This has implications, first and foremost, for small business owners and their representatives, such as the National Federation of Self Employed & Small Businesses, that campaign to sustain these ineffective commercial property reductions, and whose opinion is given and sought during government consultations, including the relatively recent Fundamental Review of Business Rates (HM Treasury, 2021).

Instead, we need to campaign for a more nuanced and targeted policy incorporating firm characteristics and local drivers and constraints. It could be more fruitful than the current generic policy based on building and land value, as our findings indicate. Having said that, the change in the commercial property fiscal regime is multi-layered and can only be achieved through a truly fundamental review, unlike the two business rates reviews concluded in 2016 and 2021 that resulted in superficial improvements with minimal effort to tackle the more pressing issues concerning the increasingly expansive relief package (Matikonis, 2022).

Broader recommendations from these findings are not to generalise or simplify the effectiveness of tax incentives. Although previous findings on tax incentives signal positive effects (Liu et al., 2019; Sterlacchini & Venturini, 2019; Venâncio et al., 2022), we show that commercial property tax reductions, which suffer from mistargeting and capitalisation, do not result in growth. This is especially relevant for the UK, which recently experienced the damaging effects of introducing poorly justified and unfunded tax incentives during the brief premiership of Liz Truss.

### Limitations and Future Work

Although the usage of the secondary data source provided a large sample size and enabled us to look at the longer-term effects, another purpose-made, possibly qualitative study of SBRR could include more specific controls and/or specifically focus on uncovering mechanisms explaining why this reduction in productivity is associated with SBRR. Alternative specifications of outcome variable would also be helpful to clarify which aspects of productivity are affected by SBRR. Furthermore, once more recent data is available, further research could also help us understand how retail, hospitality and leisure relief, used as a key tool to support businesses during COVID-19, interrelate with SBRR and affect growth.

In terms of methodology, we extend Graham and Bonner’s (2022) call to small business scholars to explore the potential of machine learning. The studies with different approaches, in different contexts and with different datasets could aid in generalisability. There are also many avenues to use more advanced techniques. For instance, future research could apply causal trees, as described by Athey and Imbens (2016), to better capture treatment effects once the method is fully developed. Further extensions for multidimensional data, such as the Least Absolute Shrinkage and Selection Operator (Belloni et al., 2014), could also be a good option.

## Supplementary Information

Below is the link to the electronic supplementary material.

Supplementary file1 (DOCX 37.7 KB)

## Data Availability

The paper uses controlled data from the UK Data Service archive, Office for National Statistics (2017a, 2017b, 2017c). The data can be obtained by filing a request directly with the UK Data Service (help@ukdataservice.ac.uk).

## Footnotes

1 We use Wooldridge’s (2009) one step GMM Total Factor Productivity estimator, which can control for simultaneity, selection and omitted price biases (Van Beveren, 2012) for our productivity estimates.

2 It is difficult to establish comparable metrics to investigate to what extent business rates burden businesses, but the burden is undoubtedly significant. This is particularly prominent amongst matched firms that did not receive SBRR and had to pay business rates (the matching process is described in Sect. 3.2). The business rates to turnover ratio averaged 10% between 2000 and 2015. The business rates to employment expenses ratio averaged 21%, and business rate expense was, on average, greater by a factor of 9 than the capital expenditure to acquire, upgrade and maintain physical assets such as property, plants, buildings, technology or equipment. Once we estimate these on raw data, the values change to 2%, 10% and 35, respectively. Note that for the raw data, the data is limited to entities that reported both units of measurement that is why it does not necessarily correspond to figures in Appendix 2.

3 See Appendix 1 for a more detailed overview of SBRR across the nations.

4 Modelling heterogeneous firms operating in heterogenous locations were already problematic within the overall BR system (Duranton et al., 2011) but this is further amplified in the SBRR context, given the previously discussed issues of mistargeting.

5 Pay As You Earn is the taxation withholding mechanism whereby employers deduct taxation from employee income on behalf of the tax authorities.

6 There are nevertheless still missing observations, particularly for smaller firms. To estimate productivity, we used the Annual Respondents Database X capital stock maintained by ONS with more details available in the user guide, supplementary to Annual Respondents Database X (Office for National Statistics, n.d.). In the same vein, before Coarsened Exact Matching, we used predictive mean matching, with the key variables of unique firm identifier, year, turnover, employment, region, sector and legal status that have almost no missing data from the Annual Respondents Database X or Business Structure Database sources. This enabled us to also include firms that may have missing observations for the particular year when matching was conducted.

7 Another alternative is instrumental variables that require strong instruments influencing specific variables, whilst we expect numerous interaction effects.

8 We report ${\mathcal{L}}_{1}$ statistic on 2004 because the vast majority of SBRR were recipients from England and thus most of them were matched on 2004 data.

9 Note that following Duranton et al. (2011), the equation could be further extended to account for heterogeneous location effects. We could denote time-invariant effects for location $j$, with ${\gamma }_{j}$, and time-variant effect for that location, ${\theta }_{jt}$. Then, the equation would become $h\left({\alpha }_{it},{Z}_{it},{f}_{i}, {\gamma }_{j},{\theta }_{jt}\right)$ but this would not change the reasoning and the location fixed effects would be captured by the establishment level estimates.

10 Scotland in 2003, England in 2005, Wales in 2007, with increase in relief in 2010.

11 The endogenous growth models of Romer (1990) and the quality ladder models of Grossman and Helpman (1991) and Aghion and Howitt (1992) theorise that innovations drive long-term economic growth and aggregate productivity.

12 Agglomeration externalities, dating back to Marshall’s (1890) ‘Industrial District-argument’, are based on asset-sharing, such as the provision of specific goods and services by specialised suppliers and the creation of a local labour market pool sustained by a local concentration of productivity, which may influence production. Spillovers arise from knowledge sharing of firms in the same sector. Feldman and Audretsch (1999) and Paci and Usai (1999) suggested using the production structure specialisation index (PS) to measure Marshallian specialisation externalities.

13 We observe similar pattern further down the branch for the regions (i.e. Group 2B), West Midlands, East and South East of England that we do not show in Fig. 4, for space reasons. A total of 869 firms in these regions between 16 and 32 years old with a PD above 0.77 have productivity greater than that for the 437 firms in areas with lower PD, although the difference is only around 2.5%.

14 The channels for this effect are diverse, from easier access to a wider selection of suppliers, secure demand or greater exposure to incremental changes which can be copied and adapted.

15 We also focus mainly on smaller firms and analyse much smaller areas that are tightly linked to a firm to better reflect their trading environments. Finally, instead of proportions, we use an inverse Gini to measure diversity and a measure of agglomeration, which places local conditions relative to the national context.
