# Supplementary material for: Small Business Property Tax Reductions and Firm Productivity
Source: Small Bus Econ (Dordr). 2023 May 9:1–18. Online ahead of print. doi: 10.1007/s11187-023-00768-0 (PMC10169169; doi:10.1007/s11187-023-00768-0)
Supplement: Supplementary file 1 — Supplementary file1 (DOCX 37.7 KB) [file 11187_2023_768_MOESM1_ESM.docx]

# Electronic Supplementary Material

**Appendix 1: Deriving SBRR**

*SBRR in England*

SBRR was derived in accordance with government publications because the Annual Respondents Database X only collects the BR each firm pays. As such, we calculate any SBRR received by calculating the firms underlying Rateable Value (RV).

$BR=RV\times m\times\left( 1-SBRR \right)$, (7)

where BR is business rates paid, RV is rateable value and m is the annual multiplier.

Defining *1- SBRR* as;

$1- SBRR=\left( 1-\left( \max- \frac{RV-int}{\frac{int}{max}} \right) \right)=1-2\times max+\frac{RV\times max}{int}$, (8)

where *max* is a maximal relief available (50% or 100% depending on the period) and *int* is the range over which the relief is paid (either £5,000 or £6,000).

Combining both equations to extract the RV, we get:

${RV}^{2}+RV\times\frac{int(1-2\times\max)}{max}-\frac{BR\times int}{max\times m}=0$ (9)

Given that maximal relief during 2005-2010 in England is equal to 0.5 *(giving 1-2×max=0)*, the equation becomes relatively straightforward to estimate:

$RV= \sqrt{\frac{BR\times int}{0.5\times m}}$ (10)

By combining the equations (11) and (13), we can now solve for SBRR in England between 2005 and 2010 from observable parameters:

$SBRR=1-\frac{BR}{m\times\sqrt{\frac{BR\times int}{max\times m}}}$ (11)

From 2010 the maximum relief was increased up to 1, so we now have to express another quadratic equation for this period:

$0={RV}^{2}+RV\times\frac{int\left( 1-2\times\max\right)}{max}-\frac{BR\times int}{max\times m}={RV}^{2}-RV\times int-\frac{BR\times int}{m}$ (12)

Combining the positive solution with the initial BR equation and solving for SBRR as a function of observable parameters, we get

$RV= \frac{int \mp\sqrt{{int}^{2}+4\times\frac{BR\times int}{m}}}{2}$ (13)

$SBRR=1-\frac{2\times BR}{m\times\left( int+ \sqrt{{int}^{2}+4\times\frac{BR\times int}{m}} \right)}$ (14)

*SBRR in Scotland and Wales*

The SBRR for other years and nations of the UK are easier to estimate because reliefs were fixed for groups of rateable values. In Wales up to 2010 (after which it aligned with England), the reliefs were retrieved in a straight forward manner as illustrated in Table 2. Whilst, Scottish reliefs were much higher than in the rest of UK and were introduced in 2003 as provided in Table 3.

**Table 2: SBRR in Wales before 2010**

| **Rateable Value (RV)** | **Year** | |
| --- | --- | --- |
|  | **2007** | **2008-2009** |
| ***RV≤2,000*** | 0.5 | 0.5 |
| ***RV≤5,000*** | 0.25 | 0.25 |
| ***RV≤6,500*** | 0 |  |
| ***RV>5,000*** |  | 0 |

*Values are in numeric expression, e.g., 0.5 corresponds to 50%.*

*Source: Non-Domestic Rating (Small Business Relief) (Wales) Orders.*

**Table 3: SBRR in Scotland**.

| Ratable Value (RV) | Year | | | | |
| --- | --- | --- | --- | --- | --- |
|  | 2003 -2004 | 2005 - 2007 | 2008 | 2009 | 2010 -2014 |
| *0≤RV≤3,000* | 0.5 | 0.5 | 0.8 | 1 | 1 |
| *3,000< RV ≤3,500* | 0.4 |  |  |  |  |
| *3,500< RV ≤4,000* |  | 0.4 |  |  |  |
| *4,000< RV ≤4,500* | 0.3 |  |  |  |  |
| *4,500< RV ≤5,000* |  | 0.3 |  |  |  |
| *5,000< RV ≤5,750* | 0.2 |  |  |  |  |
| *5,750< RV ≤6,000* |  | 0.2 |  |  |  |
| *6,000< RV ≤7,000* | 0.1 |  |  |  |  |
| *7,000< RV ≤8,000* | 0.05 | 0.1 |  |  |  |
| *8,000< RV ≤10,000* |  | 0.05 | 0.4 | 0.5 |  |
| *10,00< RV ≤11,500* |  |  |  |  |  |
| *10,000< RV ≤12,000* | 0 | 0 | 0.2 | 0.25 | 0.5 |
| *12,000< RV ≤15,000* |  |  |  |  | 0.25 |
| *15,000< RV ≤18,000* |  |  | 0 | |  |
| *18,000< RV* |  |  |  |  | 0 |

Values are in a numeric expression, e.g., 0.5 corresponds to 50%.

Source: Non-Domestic Rate (Scotland) Orders.

**Appendix 2**

**Table 4: Descriptive statistics of main variables (raw data)**

| Variable | N | Mean | St. Dev. | Pctl(25) | | Median | | Pctl(75) |
| --- | --- | --- | --- | --- | --- | --- | --- | --- |
| Industry concentration (HHI) | 6230916 | 0.01 | 0.03 | 0.002 | | 0.01 | | 0.01 |
| The Jacobian production diversity (PD) | 6230916 | 0.79 | 0.07 | 0.75 | | 0.8 | | 0.84 |
| PS | 6229677 | 1.48 | 1.8 | 0.72 | | 1.15 | | 1.73 |
| Employment | 6225925 | 14.15 | 149.28 | 1 | | 3 | | 10 |
| Turnover (‘000 £) | 6225925 | 2624.31 | 174971.5 | 72 | | 187 | | 708 |
| Business rate expense (‘000 £) | 574887 | 35.71 | 575.05 | 0 | | 1.771 | | 12.86 |
| Gross value added (‘000 £) | 528763 | 2282.48 | 134916.8 | 34 | | 157 | | 909 |
| Status | | | | | 6225887 | |  | |
| Sector | | | | | 6230916 | |  | |
| Region | | | | | 6230916 | |  | |
| Immediate | | | | | 6230916 | |  | |
| Ultimate foreign ownership | | | | | 6230916 | |  | |
| Immediate foreign ownership | | | | | 6230916 | |  | |

Source: ONS Data, Author’s Calculations. For all variables count (N) is reported and for continues variables this is supplemented by mean, median, standard deviation (SD), 25^th^ and 75^th^ percentiles.

**Appendix 3: Model Diagnostics**

The primary criticism of CART is related to overfitting (Cook and Goldman, 1984). To overcome this issue, we impose a minimum number of observations in each final node. This strategy was preferred instead of alternatives because of the disclosure controls imposed by the data owners limiting the minimum number of observations in outputs. Other options include building a model by using a training dataset and then estimating mean squared error with the hold-out data and cross-complexity pruning, estimating complexity factor to evaluate predictive capacity (James et al., 2013).

Another issue is the stability of the results (Briand et al., 2009). To test stability, Philipp et al. (2018) framework and implementation were followed. As candidate algorithms, we selected Conditional Inference Trees (Hothorn et al., 2006) that do not account for random effects and a more standard linear mixed model that accounts for random effects but assumes a more simplistic linear form. The default settings were used for all algorithms. The results are illustrated in Figure 5, which shows similarity values for all three algorithms. The REEM trees achieved the highest stability score for this dataset, with a median of 0.82.

**Figure 5: Stability (1-total variation distance) of results generated by different implementations of estimations with matched data.**

Source: ONS Data, Author’s Calculations.

To assess the accuracy, we use 10 fold validation, in which we compare the predictive capacity of our trees to other candidate algorithms. In addition to the previously used algorithms, we also include random forests known for its greater predictive accuracy. We base our strategy on Rabinowicz and Rosset’s (2020) assumptions that should be met for effective cross-validation with longitudinal data. We produce ten random subsamples where the same respondent firms are in both samples but at different times to preserve the distributional relation between the testing and training sets. For each subsample, we train the algorithms with an 80% train set and then use the remaining 20% to test the accuracy of predictions. The goal is to compare the accuracy of predictions of observations of the subjects, sampled at a random time-point from the same distribution. For the comparison, we use both Root Mean Squared Error (RMSE) and more reliable Mean Absolute Error (MAE).

The results from the k-fold validation are reported in Table 4. This table shows the means of RMSE and MAE of the four algorithms, supplemented by medians and SDs. The REEM tree achieved a relatively high accuracy when compared to alternative methods. RMSE estimates indicate that REEM tree achieved the highest accuracy, but that was not the case with MAE, which accounts for extreme outliers. Based on MAE, random forests slightly outperformed REEM tree.

.

**Table 5: Accuracy measures (MAE and RMSE) generated by different implementations of estimations with matched data.**

|  | **MAE** | | | **RMSE** | | |
| --- | --- | --- | --- | --- | --- | --- |
|  | Mean | Median | St. Dev. | Mean | Median | St. Dev. |
| **RF** | 11926.55 | 11940.23 | 523.38 | 28147.84 | 19632.10 | 12540.46 |
| **REEM tree** | 12101.39 | 11974.63 | 627.33 | 23849.07 | 19027.20 | 10155.96 |
| **ctree** | 12105.85 | 12014.93 | 528.29 | 28403.10 | 19867.47 | 12577.22 |
| **LMM** | 12128.47 | 12090.07 | 459.22 | 28392.83 | 19807.62 | 12531.69 |

Source: ONS Data, Author’s Calculations.

Given that the random forest outperformed the REEM tree in terms of RMSE, we further explore the output from this algorithm. The random forests algorithm makes multiple decision trees in parallel, and the output is the maximum voting of all the outputs from each decision tree. Table 6 reports the importance scores from the impurity-based feature importance. It orders variables based on their importance and offers an importance score that allows for comparison with a higher importance score signalling greater contribution. We see that the algorithm identified current SBRR, SBRR lagged by two, three and four as highly influential, but in feature selection, Jacobian externalities, industry concentration (HHI) and region variables have greater importance scores. This is as expected given the relatively marginal value of SBRR as input to the overall productivity. Given that the REEM tree also primarily splits on these variables, we imply that it captured relationships from most of the influential variables.

**Table 6: Random Forests Importance Scores**

| Variable | Importance score |
| --- | --- |
| PS | 2129435561322 |
| Industry concentration (HHI) | 1739529428105 |
| ID | 1649160154167 |
| Age | 1062988368526 |
| Year | 662769747910 |
| Region | 581336712563 |
| SBRR | 156688938575 |
| High growth firm (HGF) dummy | 145599059814 |
| SBRR lagged by 2 | 123988314100 |
| SBRR lagged by3 | 106464998673 |
| R&D | 90434107441 |
| SBRR lagged by 4 | 89363912658 |
| Ultimate foreign ownership | 42821004794 |
| Immediate foreign ownership | 34903013715 |

Source: ONS Data, Author’s Calculations.

**Appendix 4: Brief Introduction to Decision Trees**

Decision trees, often referred to as recursive partitioning, is a tool that builds regression models in the shape of a tree structure, illustrating possible outcomes of different decisions based on a variety of parameters. The main advantages of these mechanisms are their ability to accommodate missing data, low sensitivity to outliers, and low impact of nonlinear relationships between parameters.

The tree algorithms are based on *entropy*, defined as a top-down partitioning of data into subsets that consist of homogenous data points. The decrease in entropy after the dataset is split on an attribute is also known as *information gain,* and the splits are based on which attributes generate the highest information gain. Entropy values are calculated for every parameter that is entered into the tree model and for each decision. The parameter with the highest information gain is selected. Then the process is repeated.

The decision trees are made of two components, *leaves* and *nodes*. The former is the outcome of the decision, whilst the latter is a decision test that examines a single variable and moves to another node based on the outcome.

As a simplified example, let the parent node be noted as $S$ and the left, $L$, and right, $R$, be sub-nodes as well as y be a response value in node $S$ and $\bar{y}$ is the average response values in node $S$. The Gini index of diversity or entropy could be used to reflect the impurity of a node, which defines the node splits where each split maximises the decrease in impurity. Let G be categories in the data, $\pi_{S}(g)$ be the proportion of the observations from the G^th^ category in node S and $n_{S}$ be the number of cases in node S. The Gini index is

$I_{S}=\sum_{g=1}^{G} \pi_{s}\left( g \right)[1-\pi_{s}\left( g \right)]$ (15)

Then, after splitting into $L$ and $R$, the decrease of heterogeneity of$S$ could be defined as $\Delta I\left( S,L,R \right)=I_{S}-(I_{L}+I_{R})$. The chosen split for each node is the maximising $\Delta I\left( S,L,R \right)$. This process is repeated until the largest possible tree is obtained and no more nodes can be split.

Conditional Inference trees are a non-parametric class of decision trees that uses a statistical theory to select variables instead of selecting the variable that maximises an information measure (information gain or Gini coefficient) and thereby removes the potential bias in CART or similar decision trees.

**Appendix 5**

**Figure 6: Productivity Tree Extract of Older Firms Not Receiving Two Year Lagged SBBR, Outside of London (Group Three)**

**Figure 7: Productivity Tree Extract of Firms Not Receiving Two Year Lagged SBBR in London (Group Four)**

Productivity Tree. Extract of Group Four. It follows firms that did not receive two year lagged SBBR and were in London.

Figures in the final nodes are productivity, in terms of gross value added, and n, number of observations.

Source: ONS Data, Author’s Calculations.

References

James, G., Witten, D., Hastie, T. and Tibshirani, R. (2013) *An introduction to statistical learning with applications in R*. New York, NY: Springer.

Cook, E.F. and Goldman, L. (1984) Empiric comparison of multivariate analytic techniques: advantages and disadvantages of recursive partitioning analysis. *Journal of Chronic Diseases*, 37 (9–10), 721–31.

Briand, B., Ducharme, G.R., Parache, V., and Mercat-Rommens, C. (2009) A similarity measure to assess the stability of classification trees. *Comput. Stat. Data Anal.* 53, 1208–1217.

Philipp, M., Rusch, T., Hornik, K., and Strobl, C. (2018) Measuring the Stability of Results From Supervised Statistical Learning. *Journal of Computational and Graphical Statistics*, *27*(4), 685–700.

Hothorn, T., Hornik, K., and Zeileis, A. (2006) Unbiased recursive partitioning: A conditional inference framework. *Journal of Computational and Graphical Statistics*, *15*(3), 651–674. https://doi.org/10.1198/106186006X133933
